# Comparison of Lyme Disease in the United States and Europe

**DOI:** 10.3201/eid2708.204763

**Published:** 2021-08

**Authors:** Adriana R. Marques, Franc Strle, Gary P. Wormser

**Affiliations:** National Institutes of Health, Bethesda, Maryland, USA (A.R. Marques);; University Medical Centre Ljubljana, Ljubljana, Slovenia (F. Strle);; New York Medical College, Valhalla, New York, USA (G.P. Wormser)

**Keywords:** Lyme disease, Lyme borreliosis, *Borrelia burgdorferi*, Lyme disease spirochete, *Borrelia burgdorferi* group, Europe, United States, tick-borne diseases, vector-borne infections, bacteria

## Abstract

Lyme disease, or Lyme borreliosis, is the most common tickborne disease in the United States and Europe. In both locations, *Ixodes* species ticks transmit the *Borrelia burgdorferi* sensu lato bacteria species responsible for causing the infection. The diversity of *Borrelia* species that cause human infection is greater in Europe; the 2 *B. burgdorferi* s.l. species collectively responsible for most infections in Europe, *B. afzelii* and *B. garinii*, are not found in the United States, where most infections are caused by *B. burgdorferi* sensu stricto. Strain differences seem to explain some of the variation in the clinical manifestations of Lyme disease, which are both minor and substantive, between the United States and Europe. Future studies should attempt to delineate the specific virulence factors of the different species of *B. burgdorferi* s.l. responsible for these variations in clinical features.

Lyme disease, or Lyme borreliosis, is the most common tickborne disease in both the United States and Europe; an estimated ≈476,000 cases are diagnosed and treated per year in the United States and >200,000 cases per year in western Europe (*1*–*3*). The principal tick vector in the United States is *Ixodes scapularis*, followed by *I. pacificus*; in Europe, most cases are transmitted by *I. ricinus*, followed by *I. persulcatus* ticks (Table 1). The etiologic agent, *Borrelia burgdorferi,* was discovered in 1982 in the United States. Later, it became recognized that strains of *B. burgdorferi* in Europe were more heterogenous than strains in North America. *B. burgdorferi* sensu lato was then classified into 3 main genospecies. The originally discovered genospecies was named *B. burgdorferi* sensu stricto. The second genospecies was named *Borrelia garinii* sp. nov., and the third was named *Borrelia afzelii* sp. nov. Recently, the taxonomy of the family *Borreliaceae* (and the genus *Borrelia*) has been revised into 2 main genera, *Borrelia* and *Borreliella* (*4*). The spirochetes that cause relapsing fever retained the genus name *Borrelia,* and spirochetes that cause Lyme disease have been renamed *Borreliella* (hereafter referred to as Lyme borrelia). However, these changes have been challenged (*5*).

**Table 1:** Lyme disease in the United States and Europe

Variable	United States	Europe
Tick vector	*Ixodes scapularis*, *I. pacificus*	*I. ricinus*,* I. persulcatus*
Lyme borrelia	Mostly *Borrelia burgdorferi* sensu stricto; *B. mayonii* may occur in the upper midwestern United States	Mostly *B. afzelii* and *B. garinii*, but several other species cause human disease, including *B. burgdorferi* s.s., *B. bavariensis, B. spielmanii, and B. lusitaniae*
Speed of tick transmission of Lyme borrelia	Rarely before 36 h	*I. ricinus* ticks may transmit *B. afzelii* within 24 h
Predominant patient sex	Male patients account for 56% of reported cases during 2001–2018; no manifestation is predominant among female patients	Most cases of erythema migrans and acrodermatitis chronica atrophicans occur in women; neuroborreliosis and arthritis are predominant in men
Coinfections	Risk depends on the geographic area; the most common co-infections are anaplasmosis and babesiosis.	Risk depends on the geographic area; the most common co-infection is tick-borne encephalitis

Most cases of Lyme disease in the United States occur in the mid-Atlantic, Northeast, and Upper Midwest regions. *B. burgdorferi* s.s., which also is found in Europe, causes most human infections in the United States (*1*,*2*); the newly recognized species *B. mayonii* (which is not known to exist in Europe) is an infrequent cause of human illness in the Upper Midwest region of the United States (*6*). The incidence of Lyme disease in Europe is highest in the Scandinavian and Baltic states in northern Europe and in Austria, the Czech Republic, Germany, and Slovenia in central Europe. *B. afzelii* and *B. garinii* are the genospecies most frequently detected in *I. ricinus* and *I. persulcatus* ticks and cause most cases of Lyme disease in Europe (*1*,*2*). Neither genospecies is found in the United States. Transmission of *B. burgdorferi* s.s. by *I. scapularis* or by *I. pacificus* ticks is very infrequent during the first 36 hours after tick attachment; in contrast, transmission of *B. afzelii* by *I. ricinus* ticks may occur within 24 hours (Table 1) (*7*).

## Erythema Migrans and Other Skin Manifestations

**Figure 1:**
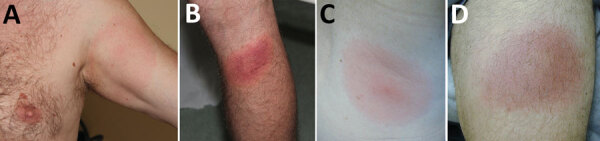
Erythema migrans skin lesions from patients in Europe (A, B) and the United States (C, D).

After Lyme borrelia are deposited in the skin by the bite of an infected *Ixodes* tick, an infection is typically established at that site, which causes the characteristic skin lesion, erythema migrans (Figure 1). Erythema migrans is the most common clinical manifestation of Lyme disease in the United States and Europe, occurring in >80% of patients in both geographic areas (*2*). Overall, US patients with erythema migrans caused by *B. burgdorferi* s.s. are less likely than patients in Europe with erythema migrans caused by *B. afzelii* or *B. garinii* to remember a tick bite at the site of the lesion (25% vs. 60% for *B. afzelii* or 64% for *B. garinii*) but more likely to have concomitant systemic symptoms (69% vs. 38% or 37%), multiple erythema migrans skin lesions (13% vs. 5% for both *B. afzelii* and *B. garinii*), and regional lymphadenopathy (29% vs. 8% or 3%) (*8*–*10*) (Table 2). Erythema migrans lesions in patients acquiring the infection in the United States have a shorter incubation period from tick bite to lesion development and are less likely to have central clearing at the time of diagnosis (*8*–*10*). The frequency of central clearing at least partially depends on the duration of the erythema migrans lesion before the diagnosis, and the duration is on average longer in Europe than in the United States (*8*–*10*). In Europe, the percentage of patients with multiple erythema migrans lesions is lower for adult patients than for children (*8*–*11*), whereas in the United States, multiple erythema migrans lesions occur with similar frequency in adults and children (*8*,*12*–*14*). Patients infected with *B. mayonii*, found in the Upper Midwest region of the United States, can exhibit multiple and very small erythema migrans lesions (*6*).

**Table 2:** Characteristics of erythema migrans in the United States and Europe

Characteristic	United States, % cases		Europe, % cases	
*Borrelia burgdorferi* sensu stricto*	*B. afzelii*†	*B. garinii*‡
Tick bite at skin site	25		60	64
Central clearing	35		69	62
Systemic symptoms	69		38	37
Multiple erythema migrans lesions	13		5	5
Regional lymphadenopathy	29		8	3

*Data from culture-confirmed erythema migrans caused by *B. burgdorferi* sensu stricto from reference (*8*). †Data combining patients with culture-confirmed erythema migrans caused by *B. afzelii* from references (*10*) and (*8*). ‡Data from culture-confirmed erythema migrans caused by *B. garinii* from reference (*9*).

**Figure 2:**
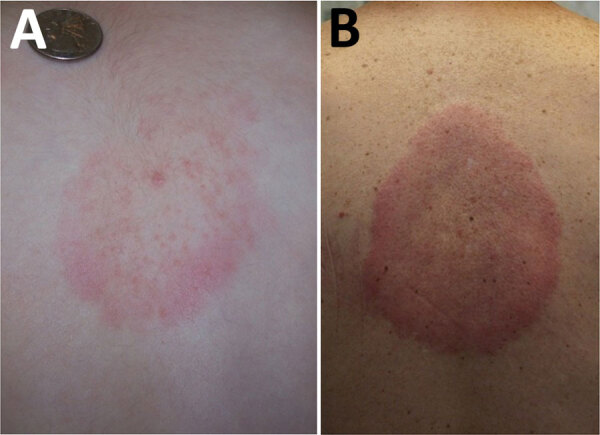
Southern tick-associated rash illness skin lesions. Adapted from Centers for Disease Control and Prevention, National Center for Emerging and Zoonotic Infectious Diseases, Division of Vector-Borne Diseases.

In the United States, an entity referred to as southern tick-associated rash illness (STARI) is associated with a skin lesion very similar to erythema migrans (Figure 2). STARI, however, occurs after the bite of ticks of a different species, *Amblyomma americanum*, and is not caused by Lyme borrelia. The etiology of STARI has not been determined. *A. americanum* ticks are most frequently found in the southeastern and south-central United States, but their range is spreading to geographic areas where *I. scapularis* tick bites are common (*15*). The potential for diagnostic confusion clearly exists in areas such as Long Island, New York, where both tick species coexist. STARI does not occur in Europe, presumably because *A. americanum* ticks are not found in that geographic area. Available, but limited, data suggest that STARI can be distinguished from erythema migrans on the basis of different serum metabolic profiles (*16*).

**Figure 3:**
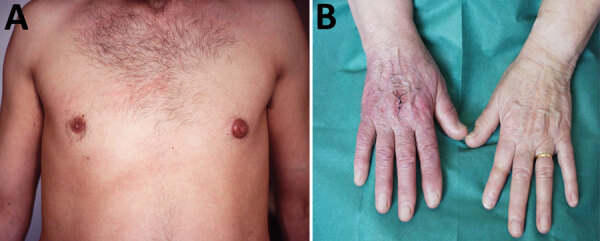
A) Borrelial lymphocytoma on nipple, showing local swelling and a remnant of erythema migrans on chest; at the time of diagnosis, the lesions had been noticed for 6 weeks. B) Acrodermatitis chronica atrophicans involving the right hand, showing red-purple discoloration, swelling, and skin atrophy; at the time of diagnosis, the lesions had been noticed for ≈2.5 years.

Two clinical manifestations of Lyme disease involving the skin occur exclusively in infections acquired in Europe: borrelial lymphocytoma and acrodermatitis chronica atrophicans (ACA) (Figure 3). Borrelial lymphocytoma appears as a small area of skin induration that slowly enlarges to a solitary bluish-red nodule or plaque with a diameter of up to a few centimeters and is predominantly located on the ear lobe in children and on the breast in adults. It usually develops at the site of a tick bite and is often accompanied with an erythema migrans lesion (*17*). ACA is a late cutaneous manifestation of Lyme disease located primarily on the extensor parts of the distal extremities. It starts with reddish-blue discoloration and swelling of the skin (an inflammatory phase), which slowly enlarges and, if untreated, is followed by atrophic changes several months to years later. For some patients, ACA was known to have been preceded by an earlier manifestation of Lyme disease, such as erythema migrans (*18*). The apparent explanation for the absence of these manifestations in the United States is that these skin infections are principally caused by *B. afzelii* (Table 3).

**Table 3:** Lyme disease clinical manifestations in the United States and Europe

Manifestation	United States	Europe
Radicular pain from Lyme neuroborreliosis	Less common in the United States*	More common in Europe
Lyme arthritis	More common in the United States in untreated patients with erythema migrans; may have septic arthritis-like presentation in children	Occurs in Europe; more commonly associated with *Borrelia burgdorferi* sensu stricto; septic arthritis-like manifestation in children seems to be rare
Acrodermatitis chronica atrophicans	No autochthonous US cases	Occurs in Europe (late manifestation)
Borrelial lymphocytoma	No autochthonous US cases	Occurs in Europe
Lyme encephalopathy	Controversial in the United States	Not recognized to occur
Diffuse axonal peripheral neuropathy	Controversial in the United States	Occurs but only in conjunction with acrodermatitis chronica atrophicans

*More studies, however, are needed.

## Neurologic Manifestations

The typical presentation of early Lyme neuroborreliosis is cranial nerve palsy, particularly facial nerve palsy, as well as lymphocytic meningitis and painful radiculitis. In the United States, the most common manifestation of early Lyme neuroborreliosis is facial palsy. Most cases of early Lyme neuroborreliosis in Europe are caused by *B. garinii* and *B. bavariensis*; in adult patients, painful meningoradiculitis is most common (*19*,*20*). In a study of 194 adult patients with Lyme neuroborreliosis in Denmark during 2015–2017, radicular pain affected 70% of the patients and facial nerve palsy 43%; intrathecal production of IgG or IgM against Lyme borrelia was found in 87% (*21*). Similar results were found in a retrospective series of 431 Lyme neuroborreliosis patients in Denmark, which included 126 children. Radicular pain (in 66%) and facial nerve palsy (in 41%) were the predominant symptoms; 84.5% of patients had evidence of intrathecal antibody production against Lyme borrelia (*22*). Although there are no comparable studies from the United States, it seems that adult US patients with early Lyme neuroborreliosis less frequently have severe radicular pain (*23*) (Table 3). Newer studies addressing Lyme neuroborreliosis in the United States would be a welcome addition for providing additional data on the frequency of particular symptoms and also on clarifying the frequency of intrathecal antibody production to *B. burgdorferi* s.s. at the time of symptom onset.

Late Lyme neuroborreliosis with encephalitis, myelitis, or encephalomyelitis has been reported in Europe but is very rare in the United States (*24*). On the other hand, 2 neurologic manifestations that have been reported to occur in the United States are now regarded as controversial. The first is Lyme encephalopathy, a poorly defined entity, which occurs in the absence of cerebrospinal fluid pleocytosis, intrathecal production of antiborrelial antibody, or direct microbiologic evidence of *B. burgdorferi* s.s. infection in the central nervous system. Symptoms include memory and concentration complaints. A now-recognized source of confusion with regard to this entity is that some patients with posttreatment Lyme disease syndrome in the United States report cognitive difficulties, and a subset of these patients have abnormal neurocognitive test results (*25*,*26*). Adding to the controversy, however, is the question of what constitutes dysfunction on such testing and the clinical significance of the test results (*27*).

The second controversial neurologic manifestation in the United States is a chronic distal symmetric sensory neuropathy. In Europe, distal axonal neuropathy in the context of Lyme disease is exclusively associated with ACA. In patients with ACA, the neuropathy is predominantly sensory, most often in the involved skin areas (*28*). Case series in adult patients in the United States reported a similar neuropathy but without evidence of ACA (*29*,*30*). The distribution of neurologic deficits, which is predominantly sensory, is distal and typically symmetric, but it can be asymmetric. The neuropathy is primarily axonal and thought to be a mononeuropathy multiplex, which can be confluent (*24*). Cerebrospinal fluid examination is usually unremarkable. Major concerns have been raised as to whether this entity has been appropriately validated as a manifestation of *B. burgdorferi* s.s. infection in the United States (*31*).

Overall, several factors have probably contributed to the belief that cognitive complaints or a chronic distal symmetric sensory peripheral neuropathy was attributable to Lyme disease in the United States (*31*). These factors include the use of diagnostic testing that is no longer considered valid, failure to appreciate that background seropositivity for antibodies to *B. burgdorferi* s.s. exists, and failure to include matched controls to determine if an association with cognitive complaints or peripheral neuropathy with a positive diagnostic assay for Lyme disease is higher than expected.

## Lyme Arthritis

Lyme arthritis will develop in ≈60% of US patients with untreated erythema migrans over a 2-year period (*32*) and is said to comprise 28% of Lyme disease cases reported to the US Centers for Disease Control and Prevention that have data on symptoms available (*33*). Lyme arthritis seems to be less frequent in Europe (*34*,*35*), and for untreated patients in Europe, the interval between onset of erythema migrans and development of Lyme arthritis may be shorter (*1*). Of note, *B. burgdorferi* s.s. was the most prevalent species of Lyme borrelia found in synovial fluid in a study of patients with Lyme arthritis in Europe (*36*). An acute manifestation of Lyme arthritis in children in the United States can mimic septic arthritis; this manifestation, however, does not seem to occur in children in Europe with Lyme arthritis (*37*).

With regard to demographics, Lyme disease in the United States is more common in male patients (56% of the patients reported during 2001–2018 were male) (*38*). Indeed, no clinical manifestation has been associated with a female predominance in the United States, whereas in Europe, most cases of erythema migrans and ACA occur in women (*39*,*40*). Many studies (but not all) demonstrated a male predominance for Lyme neuroborreliosis and Lyme arthritis (*22*,*35*,*36*,*41*).

## Laboratory Diagnosis, Treatment, and Prophylaxis

**Figure 4:**
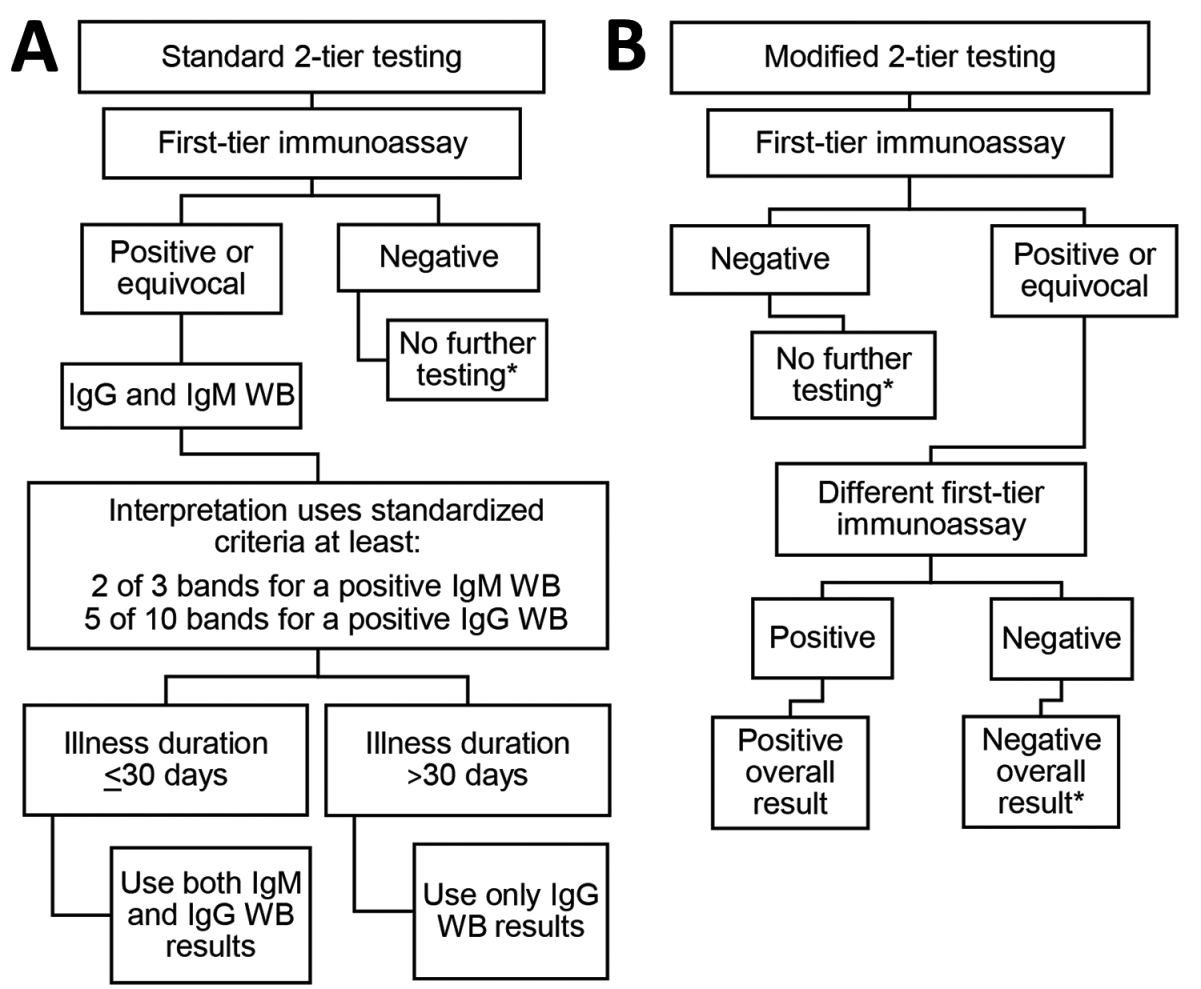
Standard 2-tier and modified 2-tier algorithms for serodiagnosis of Lyme disease. The US Centers for Disease Control and Prevention recommended a standard 2-tier algorithm (A) and the modified 2-tier algorithm (B). *For patients with signs or symptoms consistent with Lyme disease for <30 days, the provider may treat and follow up with a convalescent-phase serum sample. Patients with erythema migrans should receive treatment on the basis of the clinical diagnosis. WB, Western blot.

In the United States and Europe, most laboratory tests performed to diagnose Lyme disease are based on detecting serum antibodies to Lyme borrelia. Because Lyme disease in Europe is caused by a more diverse group of Lyme borrelia, criteria for test interpretation were more challenging to standardize than in the United States. In the United States, the Centers for Disease Control and Prevention has recommended the standard 2-tier algorithm since 1995. This approach typically uses a sensitive enzyme immunoassay (EIA) as the initial step. A negative result requires no further testing. A positive or equivocal result is followed by supplemental testing using separate IgM and IgG immunoblots as the second-tier assay. The interpretation of immunoblot results uses standardized criteria (at least 2 of 3 signature bands for a positive IgM immunoblot and 5 of 10 signature bands for a positive IgG immunoblot). Results from the IgM immunoblot are only relevant when the duration of the illness is <30 days. Of note, testing performed in Europe is more likely to have positive results for patients who acquired Lyme disease in the United States than is testing performed in the United States to diagnose infection acquired in Europe (*42*). Recently, a 2-EIA approach has been approved as an alternative (or modified) 2-tier testing strategy (Figure 4). This new approach has higher sensitivity in early disease, similar specificity (*43*), greater ability for automation, and offers objective, quantitative values that leads to less variability in interpretation of the result. Also, the 2-EIA approach can be used in the United States and Europe. Moreover, it opens the door for a possible point-of-care test, a development that would be particularly helpful for patients with facial palsy, carditis, and pediatric patients with Lyme arthritis when septic arthritis is part of the differential diagnosis. A disadvantage is that the 2-EIA approach does not establish the extent of IgG seropositivity, which is essential knowledge for diagnosing late Lyme disease.

Recommendations for treating of Lyme disease are generally very similar in guidelines for the United States and Europe. One difference is that phenoxymethylpenicillin (penicillin V) is recommended for treatment of erythema migrans and borrelial lymphocytoma by some of the guidelines in Europe but is not part of the treatment recommendations in the United States (*1*,*44*,*45*). Another difference is the recommendation by some authorities in Europe to use intravenous ceftriaxone to treat erythema migrans, as well as other manifestations of Lyme disease, in pregnant women; whereas in the United States, antimicrobial drug treatment of Lyme disease for pregnant women is the same as that for nonpregnant patients, except that doxycycline is not recommended for pregnant women (*1*,*44*,*46*). Postexposure antimicrobial prophylaxis with a single 200-mg dose of doxycycline has been shown to reduce the risk for Lyme disease after an *I. scapularis* tick bite and is recommended for consideration for tick bite prophylaxis in the United States (*44*). A recently published study conducted in Europe has also shown that a single 200-mg dose doxycycline successfully prevented Lyme disease after a tick bite (*47*). To what extent doxycycline will be used in Europe after a tick bite is unknown; the standard of care has been observation (*1*).

## Lyme Borrelia Co-infections

*Ixodes* ticks can carry multiple pathogens, and a single tick bite may result in transmission of >2 infectious agents. Pathogens potentially transmitted by *I. scapularis* ticks to humans include *B. burgdorferi* s.s*.*, *B. mayonii*, *B. miyamotoi*, *Anaplasma phagocytophilum*, *Babesia microti*, *Ehrlichia muris eauclairensis*, and the deer tick virus subtype of Powassan virus (*48*). The frequency of co-infections depends on the prevalence of the infectious agents in ticks, which will vary in different geographic areas. In the United States, *A. phagocytophilum* and *B. microti* are the most frequent co-infections in patients with Lyme disease (*49*). In the northeastern United States, ≈11% of patients infected with *B. miyamotoi*, a relapsing fever spirochete, are co-infected with *B. burgdorferi* s.s.; of note, *B. miyamotoi* infections per se can cause positive results on first-tier tests for Lyme disease, potentially leading to diagnostic confusion. Encephalitis caused by deer tick virus is relatively rare, but cases may be increasing. In Europe, in addition to Lyme borrelia, *I. ricinus* ticks can transmit tick-borne encephalitis virus, *A. phagocytophilum*, species of the bacterial genus *Rickettsia*, *B. miyamotoi*, and *Babesia* protozoans. Tick-borne encephalitis virus is well recognized as a cause of co-infection in patients with Lyme disease in Europe (*50*). More data are needed on the frequency of co-infections in both the United States and Europe.

## Conclusions

Lyme disease is common in many areas of the United States and Europe and may have a variety of clinical manifestations. The duration of infection and the species of Lyme borrelia causing the infection can affect the clinical features of Lyme disease. In the United States, patients with erythema migrans more often have concomitant systemic symptoms than do patients in Europe. In Europe, Lyme arthritis is associated with *B. burgdorferi* s.s. and Lyme neuroborreliosis with *B. garinii*. Certain cutaneous manifestations of Lyme disease in Europe do not occur at all in the United States. It will be valuable to delineate the specific virulence factors of the different species of Lyme borrelia that contribute to these clinical differences.
